# Sexual dimorphic impacts of systemic vincristine on lower urinary tract function

**DOI:** 10.1038/s41598-022-08585-3

**Published:** 2022-03-24

**Authors:** Nao Iguchi, Sarah L. Hecht, Dexiang Gao, Duncan T. Wilcox, Anna P. Malykhina, Nicholas G. Cost

**Affiliations:** 1 Division of Urology, Department of Surgery, University of Colorado Denver School of Medicine, Aurora, CO 80045 USA; 2 Department of Pediatric Urology, Doernbecher Children’s Hospital, Oregon Health & Science University, Portland, OR 97239 USA; 3 Department of Pediatrics, University of Colorado Denver School of Medicine, Aurora, CO 80045 USA; 4 Children’s Hospital Colorado, 13123 E. 16th Avenue, Aurora, CO 80045 USA

**Keywords:** Cancer, Molecular biology, Diseases, Medical research, Oncology, Pathogenesis, Risk factors, Signs and symptoms, Urology

## Abstract

Vincristine (VCR) is one of the most common chemotherapy agents used in pediatric oncology. Despite the well-known VCR-induced peripheral neuropathy, potential impacts of VCR on lower urinary tract (LUT) function remain poorly defined. We investigated the effects of systemic VCR exposure in childhood on LUT function by using juvenile mice treated with VCR (4 mg/kg) or saline and evaluated at 5 weeks later. VCR induced a decreased urinary frequency with increased functional bladder capacity and non-void contractions. There were no changes in detrusor contractility between the groups. VCR exposure caused sexual dimorphic changes; in females, increased intravesical pressure at micturition and downregulations of a major player in bladder afferent firing, Htr3b, in the bladders, and *Cav1.2* in the lumbosacral dorsal root ganglia (Ls-DRG), while male mice displayed increases in bladder compliance and detrusor activity, upregulations of IL-2, Trpa1 and Itga1 in the bladders and neuroinflammation-related genes, *P2*×*4*, *P2*×*7*, *IL-2* and *CD68* in the Ls-DRG. These results suggest that that systemic VCR exposure caused sensory neuropathy via sex-dimorphic mechanisms, leading to altered LUT function. These changes might clinically present as gender-specific signs or symptoms of LUT dysfunction, and follow-up urological assessment may be of benefit for pediatric cancer patients treated with VCR.

## Introduction

Vincristine (VCR) is frequently used in multi-agent chemotherapy regimens to treat a variety of childhood cancers. However, VCR may cause dose-limiting neurotoxicity. Many classes of chemotherapy agents including platinum derivatives (oxaliplatin and cisplatin); taxanes (docetaxel and paclitaxel); vinca alkaloids (vincristine); thalidomide and bortezomib, are known to cause peripheral neuropathy through different pathophysiological mechanisms^1^. This condition is termed chemotherapy-induced peripheral neuropathy (CIPN), and the risk of developing CIPN increases with higher doses, multiple cycles of therapy, and combinations of neurotoxic chemotherapy.

VCR binds to the β-subunit of tubulin and inhibits the addition of new tubulin subunits to microtubule ends, thus, leading to microtubule depolymerization. In dividing cells, this VCR-induced disruption of microtubule aggregation in the miotic spindle leads to mitotic arrest and cell death. Neurons also depend on microtubules to maintain the structure of axons and dendrites and serve as tracks for intracellular trafficking by motor proteins to transport specific cargoes including proteins, synaptic vesicles, mitochondria, and other organelles. Such disruption of axonal transport is considered as the primary mechanism of VCR-induced peripheral neuropathy (VIPN). Other potential mechanisms of VIPN include activation of the immune system and subsequent neuroinflammation, as well as mitochondrial dysfunction resulting from perturbation of calcium homeostasis^1^. VIPN is clinically characterized primarily by numbness, tingling, and a painful sensation felt in the hands and feet, muscle weakness, gait abnormalities, and constipation due to its effects on the sensorimotor as well as autonomic nerves^1^. Normal lower urinary tract (LUT) function requires coordinated interaction of the autonomic and somatic nervous systems^2^. Perturbation of these neural systems could impact LUT function, especially during childhood when neurons are actively elongating their axons and dendrites. Despite widespread use of VCR in both pediatric and adult patients with cancer, the potential impact of VCR on LUT function has been poorly defined. A recent survey study of childhood cancer survivors from our group has implicated VCR exposure in LUT dysfunction (LUTD) with differences observed between male and female patients^3^. This study aims to examine how VCR exposure affects LUT physiology and function by studying a juvenile murine model.

## Materials and methods

All experimental protocols were reviewed, approved by our Institutional Animal Care and Use Committees (IACUC, approval # 00441). All experiments were performed in accordance with NIH guidelines and regulations, as well in compliance with the ARRIVE guidelines 2.0 (https://arriveguidelines.org/arrive-guidelines). Mice were maintained in the animal facility (14-h light: 10-h dark cycle) with free access to water and chow.

### Animals

**Figure 1:**
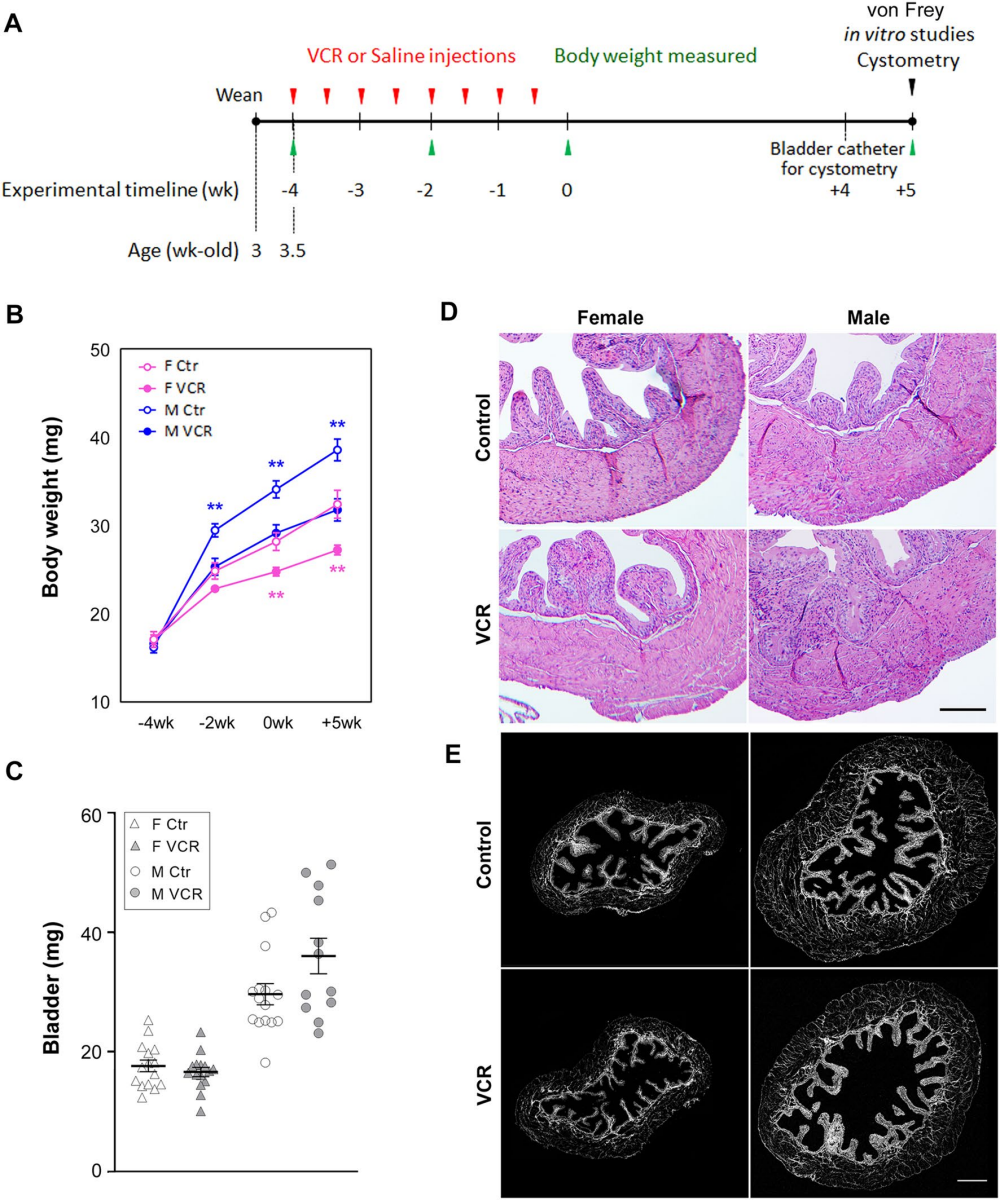
Comparison of changes in body weights, bladder weights and histology between VCR-administered and the control groups. (**A**) experimental timeline. (**B**) body weight was measured on the day before the first (−4wk) and fifth (−2wk) administration, the end of administration cycles (0wk) and 5 weeks after the last, 8th administration. (**C**) bladder weight measured at 5 weeks after the last administration of agents. Open and grey triangles indicate female mice in the control and VCR groups. Open and grey circles indicate male mice in the control and VCR groups [N = 12–15 per group per sex] Mean ± SE. **p < 0.005 vs. the control mice. Representative images of H&E staining (**D**) and collagen distribution (grey) in the bladder by SHG imaging (**E**). Scale bars = 400 µm. Figures were prepared using Adobe Photoshop CS6 and GraphPad Prism 8.4.3 (https://www.graphpad.com/scientific-software/prism/).

CD-1 mice (Charles River Laboratories, Hollister, CA, USA) were given a dose of 0.5 mg/kg of body weight of vincristine sulfate injection USP (Hospira, Lake Forest, IL, USA) (VCR group, 25 females and 30 males) or the same volume of sterile saline (control group, 22 per sex) by intraperitoneal injections twice a week for 4 weeks (cumulative VCR doses of 4 mg/kg) starting at 3.5-week-old. Three female and eight male mice were excluded from the study after 2–4 weeks of VCR administration due to lethargy or distress not relieved by our IACUC-approved regimen. All experiments using the two groups of mice were conducted at 5 weeks after the last administration cycle (Fig. 1A). Number of animals used in each experiment was listed in the Supplementary Table S2. ChAT-Cre^+/−^:: ZsGreen^+/−^ mice (2 females and 1 male) were obtained from breeding between two transgenic mouse strains, B6;129S6-Chat^tm2(cre)Lowl/J^ and B6.Cg-Gt(ROSA)26Sor^tm6(CAG-ZsGreen1)Hze/J^ (Jackson Laboratory, Bar Harbor, ME, USA, Stock No. 006410 and 007906).

### Cystometry

A subset of mice underwent surgical catheter implantation in the bladder for cystometry studies at 4 weeks after the last administration cycle as previously described^4^. Briefly, a flared-end polyethylene catheter (PE-10) was inserted through a puncture on the bladder dome and secured with a 7-0 Prolene suture (Ethicon, Somerville, NJ, USA). The catheter was tunneled subcutaneously and exteriorized at the scapula region and secured the purse-string suture around the catheter. The catheter was filled with sterile saline after confirming no leakage at the bladder and the free end was sealed. The abdominal incision was closed, and animals received a single dose of bupivacaine (2 mg/kg) and penicillin (40,000 IU/kg) subcutaneously. A single dose of carprofen (5 mg/kg) was given subcutaneously daily from 0 to 3 days after surgery. Awake cystometry was performed during daytime (10 a.m. to 4 p.m.) at 1 week after the bladder catheter implantation surgery. The tip of the exteriorized bladder catheter located at the base of the mouse neck was connected to a pressure transducer and an infusion pump of the cystometry station (Med Associates, St. Albans, VT, USA). Room temperature saline was infused into the bladder at the rate of 15 μl/min (N = 4–6 per group per sex). Each animal was observed for six voiding cycles of reproducible micturition patterns. Urodynamic values were recorded continuously during testing, and four parameters; maximum intravesical pressure at micturition, functional bladder capacity, voided volume, and the number of non-void contractions (NVCs) were analyzed using Cystometry Analysis Software (SOF-552, Med Associates). The NVCs were defined as intravesical pressure rises greater than one-third of average maximal voiding pressure in each animal without triggering micturition.

### Manual von Frey tests

To evaluate and standardize the degree of VIPN in our murine model, manual von Frey tests were conducted (N = 3–7 per group per sex) as previously described^5^. Animals were individually placed in clear plexiglass chambers with a metal grid floor allowing access to their plantar surface and were allowed to acclimatize prior to the start of the experiment. The von Frey hairs (0.04–2 g) were applied to the plantar surface of the hind paw with enough force to allow the filament to bend. The stimulus was repeated ten times at intervals of several seconds on each side of hind paw. The animals were subjected to a set testing once per day for two consecutive days. A positive response was noted if the paw was sharply withdrawn or if the mouse flinched upon removal of the hair.

### Histological analysis

Urinary bladders from mice (N = 4–5 per group per sex) were harvested at 5 weeks after the last injection cycle, fixed with 4% paraformaldehyde for at least 48-h at 4 °C, and embedded in paraffin. Five-micrometer sections were cut, mounted on slides, and then subjected to hematoxylin and eosin (H&E) staining and collagen fiber imaging with a second harmonic generation (SHG) microscopy at 100× magnification (Carl Zeiss Microscopy, LLC, Thornwood, NY, USA)^6^. The H&E sections were examined and image-captured at 40× magnification under a microscope (CH-2, Olympus, Tokyo, Japan). Areas of whole tissue section, detrusor smooth muscle (DSM) layer*,* and collagen fibers (pseudo colored in grey) in SHG images of each section were measured by using Adobe Photoshop (Adobe Systems Inc., San Jose, CA, USA).

### In vitro detrusor contractility measurements

In vitro DSM contractility measurements were performed as previously described^7^. Briefly, freshly isolated urinary bladders from mice in each group at 5 weeks after the last injection cycle (N = 6 per group per sex) were cut into 2 halves longitudinally. Each strip (~ 4 mm × 6 mm) was placed in organ baths (Radnoti, Monrovia, CA, USA) filled with oxygenated Tyrode’s buffer (in mM; 125 NaCl, 2.5 KCl, 23.8 NaHCO_3_, 0.5 MgCl_2_, 0.4 NaH_2_PO_4_, 1.8 CaCl_2_, and 5.5 glucose) at 37 °C. Tissues were equilibrated for 30 min, and then stretched to their optimum length for muscle contraction (L_o_) in which the maximum force for muscle contraction produced by electrical field stimulation (EFS; 70 V, 32 Hz). Once L_o_ was determined, each muscle strip was equilibrated for 30 min in fresh Tyrode’s buffer, and then spontaneous contractions in the bladder strips were collected for 2 min. Each bladder strip was subjected to contractile evaluation in response to EFS (70 V, 2–32 Hz), carbachol (CCh, 1 to 100 µM), α,β-methylene ATP (αβMeATP, purinoceptor P2rx agonist, 45 µM) and high KCl (125 mM replaced NaCl in Tyrode’s buffer). Contractile responses to EFS (32 Hz) were also recorded after 20 min of incubation with the following substances: (1) αβMeATP, and the combination of (2) αβMeATP and atropine (1 µM). Peak force of the contractile response was calculated in grams of tension per weight of individual bladder strip. Contractile parameters were measured using PowerLab Lab-Chart version 8.1.9 (AD instruments, Colorado Springs, CO, USA). Force measurements were performed and analyzed as previously described^7^.

### Quantitative real-time polymerase chain reaction (qPCR)

Total RNA isolated from the urinary bladders and Ls-DRG (L1-S2) from each group of mice (N = 4–5 per group per sex) using QIAzol (QIAGEN, Hilden, Germany), was transcribed into cDNA using iScript cDNA kit (Bio-Rad, Hercules, CA, USA), which was used in qPCR using LightCycler 96 system and LightCycler SYBR Green Master Mix (Roche Molecular Systems, Pleasanton, CA, USA)^7^. Primer sequences used for qPCR are listed in the Supplementary Table S1. Relative expression of mRNA levels of each transcript was quantified using the 2^−ΔΔCT^ method. The bladder and Ls-DRG data were normalized to the mean of 2 housekeeping genes, glyceraldehyde 3-phosphate dehydrogenase (*Gapdh*) and TATA-binding protein (*Tbp*) or phosphoglycerate Kinase 1 (*Pgk1*), respectively.

### Western blotting

Bladders (N = 3 per group per sex) were lysed in T-PER reagent (invitrogen, Waltham, MA, USA) supplemented with cOmplete proteinase inhibitor cocktail (Roche), and centrifuged for 10 min at 4 °C. The supernatant was collected, and the protein concentration was determined by Bradford assay (Bio-Rad). Protein samples subjected to 7, 10 or 12% SDS–polyacrylamide gel electrophoresis and transferred to a nitrocellulose membrane (Bio-Rad). Blots were blocked with 5% skim milk in Tris-buffered saline supplemented with 0.1% Tween 20 (TBST) and washed with TBST. Blots were incubated at 4 °C overnight with primary antibodies (Table 1A) in 5% BSA in TBST. Blots were washed with TBST and incubated with secondary antibodies (Table 1B) for 1 h at room temperature and washed with TBST. The immunoreactivities were detected by fluorescence or using ECL substrate (Thermo Fisher Scientific, Waltham, MA, USA). The protein-specific signals were measured using Fiji ImageJ software (Version 1.53c, National Institutes of Health, Bethesda, MD, USA). The signals specific for each antibody were quantified using ImageJ software and normalized to the signal for Gapdh*.*

**Table 1:** Antibodies.

A. Primary antibodies				
	Vendor and catalog no.	Application and dilution	Host species	Validation (DOI)
Desmin	Novus Biologicals (Littleton, CO, USA), NB120-15200	1:400 (IF)	Rabbit	(10.1152/ajprenal.00090.2019)
Gapdh	Proteintech (Rosemont, IL, USA), HRP-60004	1:5,000 (WB)	Mouse	(10.1038/s41598-020-80794-0)
Htr3b	Alomone Labs (Jerusalem, Israel), ASR-032	1:100 (IF), 1:500 (WB)	Rabbit	Fig. S2A
Itga1	Thermo Fisher Scientific, PA579525	1:200 (IF), 1:1000 (WB)	Rabbit	Fig. S2A
IL-2	St John's Laboratory (London, UK), STJ93688	1:1,000 (WB)	Rabbit	Fig. S2A
PKC_Ɛ_	Santa Cruz Biotechnology (Dallas, TX, USA), sc-214G	1:500 (WB)	Goat	(10.4049/jimmunol.1102985)
Trpa1	Santa Cruz Biotechnology, sc-376495	1:100 (IF)	Mouse	(10.1111/bph.15467)
β-Tubulin	Proteintech, HRP-66240	1:5,000 (WB)	Mouse	Fig. S1
Tubb3	Proteintech, CL594-66375	1:250 (IF)	Mouse	Fig. S2B

B. Secondary antibodies				
	Conjugate	Vendor and catalog no.	Application and dilution	
Goat IgG	DyLight800	Invitrogen, SA510092	1:5000 (WB)	
Mouse IgG	Starbright 700	Bio-Rad Laboratories, 12005870	1:5000 (WB)	
	FITC	Jackson ImmunoResearch (West Grove, PA, USA), 115-095-003	1:10,000 (IF)	
Rabbit IgG	Starbright 520	Bio-Rad Laboratories, 12005870	1:5000 (WB)	
	FITC	Jackson ImmunoResearch, 111-095-003	1:5000 (IF)	
	Cy3	Jackson ImmunoResearch, 111-165-003	1:10,000 (IF)	

### Immunofluorescence labeling

Paraformaldehyde-fixed paraffin-embedded bladder sections (5 µm thickness) (described in “Histological analysis”) were subjected to heat-induced antigen retrieval (10 mM Tris, 1 mM EDTA, and 0.05% Tween 20, pH 9.0)^8^. Then, sections were rinsed with phosphate buffered saline (PBS) and blocked with 3% normal goat serum (w/v) in PBS supplemented with 0.1% Tween 20 (PBST) for 1 h at room temperature. The tissues were then incubated with primary antibodies (Table 1A) diluted in 3% normal goat serum in PBST overnight at 4 °C. After three times washes with PBST for 10 min each, the sections were incubated with a secondary antibody (Table 1B) diluted in 3% normal goat serum in PBST for 1 h at room temperature. Then sections were washed three times for 10 min each with PBST, mounted with Flouroshield containing DAPI (Abcam, Cambridge, UK) and covered with glass slides. Control experiments performed without primary antibodies showed neither non-specific labeling nor cross-reactivity between secondary antibodies. The stained sections were examined and image-captured at 200× magnification by Zeiss ZEN2011 software (https://www.zeiss.com/microscopy/us/products/microscope-software/zen.html) and Zeiss LSM780 microscope (Zeiss). The area and intensity of immunoreactive (IR) signals with each antibody were measured using Adobe Photoshop CS6 following the software’s instruction (https://helpx.adobe.com/photoshop/using/measurement.html)^9^. All measurements were conducted in a blind fashion to avoid biased interpretation. Three sections at least 100 µm apart from each other from at least 3 animals per sex per group were analyzed for reproducibility. Bladder sections from ChAT-Cre^+/−^:: ZsGreen^+/−^ mice (N = 3) were used to identify choline acetyltransferase (ChAT)-expressing cells.

### Statistical analysis

All data were analyzed using two-way ANOVA among 4 groups (each sex × treatment (control and VCR) groups) using GraphPad Prism 8.4.3 (GraphPad Software, La Jolla, CA, USA). GraphPad outlier calculator (GraphPad Software) was used to detect outliers, which were excluded from the analysis in in vitro detrusor contractility measurements. Treatment differences of significance by F test from ANOVA were analyzed by two-tailed unpaired t-test for each sex using GraphPad Prism 8.4.3. A probability value of p < 0.05 was regarded as significant. Results are expressed as means ± standard error of the mean (SE).

## Results

### Systemic VCR did not affect bladder morphology in mice

A significant growth deficit was observed in both female and male mice after receiving VCR of a cumulative dose of 2 or 4 mg/kg of body compared with the control group (Fig. 1B). Male mice in VCR group showed a trend of increased bladder wet weight compared to the control group (36.0 ± 3.0 vs. 29.6 ± 1.8 mg, p = 0.06), but not in female mice (16.6 ± 0.8 vs. 17.6 ± 1.0 mg, p = 0.43) (Fig. 1C). There were no apparent differences in the bladder morphology between the control and VCR-treated groups in terms of the structure of the urothelial, lamina propria and DSM layers, and the distribution and proportional amount of collagen contents (Fig. 1D,E).

### Systemic VCR exposure induced sexual dimorphic changes in urodynamic parameters

**Figure 2:**
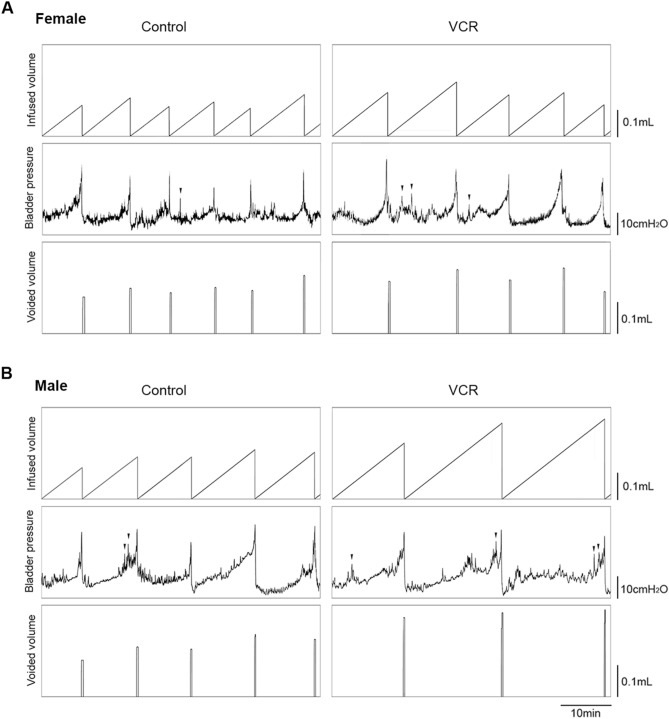
Lower urinary tract function analyses in cystometry. Representative cystometrogram traces from unanesthetized, unrestrained female (**A**) and male (**B**) mice in control (left panels) and VCR groups (right panels) during a continuous intravesical infusion of room temperature saline. Volume infused (top panels), intravesical pressure (middle panels) and voided volume (bottom panels) are shown. Arrowheads in the intravesical pressure traces indicate non-voiding bladder contractions. Figures were prepared using Cystometry Analysis Software (SOF-552, https://www.med-associates.com/product/cystometry-analysis-data-analysis-software/) and Adobe Photoshop CS6.

Three urodynamic parameters; functional bladder capacity (infused volume of saline in each micturition cycle), volume of voids, and NVCs were significantly increased in VCR group compared to the control group in both sexes of mice (Fig. 2 and Table 2). All groups of mice had approximately 100% of the bladder voiding efficiency (the volume voided/infused in each micturition cycle). VCR exposure induced a notable increase in the maximal intravesical pressure at micturition (Pves max) in females but not in males compared to that in the control group. Male mice exposed to VCR treatment showed increased bladder compliance (bladder capacity/change in intravesical pressure in each micturition cycle) to that in mice treated with saline, but females did not show any difference between the groups.

**Table 2:** Comparison of urodynamic parameters between the groups**.**

Sex	Treatment [# animals]	Infused (µl)	Voided (µl)	Efficiency (%)	Pves max (cmH_2_O)	Compliance (µl/cmH_2_O)	NVCs (cycle^−1^)
F	Saline [4]	133 ± 9	131 ± 9	100 ± 3	20.5 ± 1.3	6.8 ± 0.4	0.1 ± 0.1
	VCR [5]	168 ± 10*	167 ± 10*	100 ± 2	25.4 ± 0.9**	6.8 ± 0.5	0.8 ± 0.2**
M	Saline [6]	163 ± 12	162 ± 11	99 ± 2	22.8 ± 1.1	7.2 ± 0.3	0.5 ± 0.2
	VCR [6]	246 ± 19**	248 ± 18**	102 ± 2	21.7 ± 1.0	11.3 ± 0.7**	1.4 ± 0.3*

Significant increases in functional bladder capacity, voided volume, and the number of non-void contractions were observed in both female and male mice received VCR. VCR exposure induced increases in Pves max and bladder compliance in females and males, respectively, in comparison with the control group. Mean ± SE, *p < 0.05 and **p < 0.005 vs. the control group, Pves max, maximum intravesical pressure at micturition, NVCs, non-void contractions per micturition cycle.

### VCR exposure induced mechanical hyposensitivity

**Figure 3:**
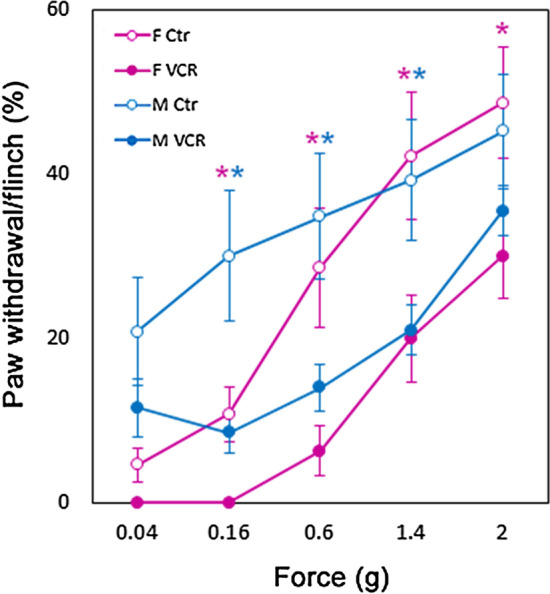
VCR exposure induced mechanical hyposensitivity in mice. Pink and blue lines and asterisks represent female and male mice, respectively. Open and filled circles represent the control and VCR groups, respectively. Mean ± SE, *p < 0.05 between two groups. Figures were prepared using GraphPad Prism 8.4.3 (https://www.graphpad.com/scientific-software/prism/).

Manual von Frey tests revealed that VCR exposure led to a significant decrease in the paw withdrawal or flinching responses in both sexes of mice compared to those in the control groups (Fig. 3). The mechanical hyposensitivity appeared to be more severe in female mice than in male mice who received VCR.

### VCR induced detrusor overactivity in male mice

**Figure 4:**
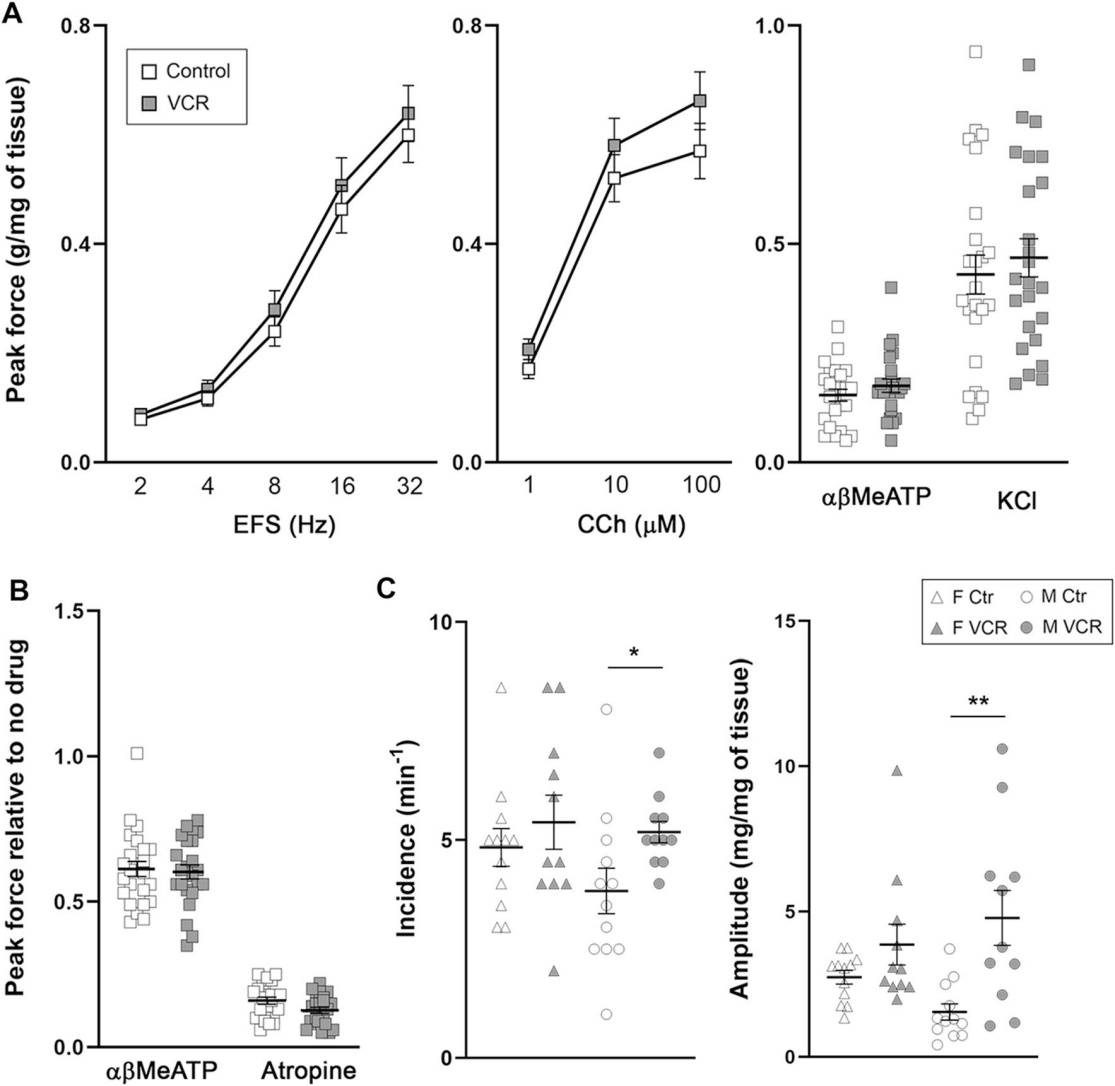
In vitro physiological evaluation of the bladder strips. **A**, peak contractile force in response to electric field stimulation (EFS, left), carbachol (CCh, center), and αβ-MeATP and KCl (right) [N = 6, 12 bladder strips per group per sex]. **B**, peak contractile force in response to EFS at 32 Hz after preincubation of αβ-MeATP and atropine. The force was normalized with tissue weight and expressed as relative to the response to EFS in absence of inhibitors. Open and grey boxes represent the control and VCR group, respectively. **C**, the amplitude, and the frequency of spontaneous contractions of the bladder strips. Open and grey triangles represent female mice in the control and VCR groups, respectively [12 bladder strips per group]. Open and grey circles represent male mice in the control [12 bladder strips] and VCR [11 bladder strips] groups, respectively. Mean ± SE, *p < 0.05, **p < 0.005 vs. the control group. Figures were prepared using Adobe Photoshop CS6 and GraphPad Prism 8.4.3 (https://www.graphpad.com/scientific-software/prism/).

There were no changes in contractile responses to all stimuli tested; EFS, CCh, αβMeATP and KCl between two groups (Fig. 4A). Both female and male mice showed a similar pattern of contractility evoked by these four types of stimulation between the groups. There were also no changes in the contractility triggered by EFS at 32 Hz following the preincubation of bladder strips with αβMeATP which desensitizes purinoceptors, and atropine, a muscarinic receptor agonist (Fig. 4B). Bladder strips from male mice received VCR revealed a significant increase in spontaneous contractions for both frequency and amplitude compared to the control group but not in females (Fig. 4C), suggesting that VCR induced male-specific detrusor overactivity.

### VCR induced sexually dimorphic changes in gene expression patterns in the bladders and Ls-DRG

**Figure 5:**
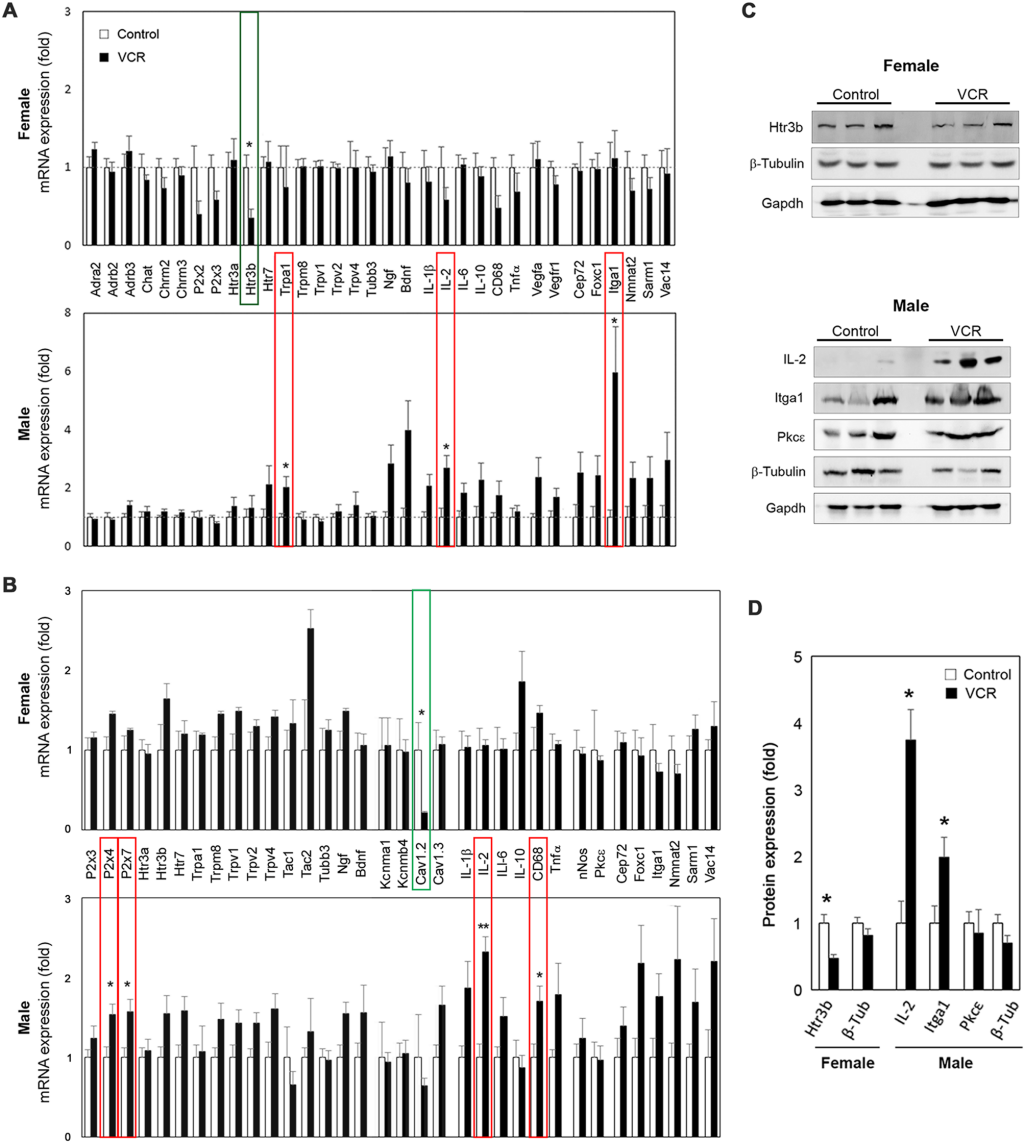
Gene expression analyses. The level of mRNA expression of each gene in the bladder (**A**) and Ls-DRG (**B**) is expressed as the fold difference to that in the control group. (**A**) normalized with a mean value of *Gapdh* and *Tbp* [N = 4–5 per group per sex] (**B**) normalized with *Pgk1*. White and black bars represent the control and VCR groups, respectively. The upper and lower panels show the data for female and male mice, respectively. Mean ± SE. Representative Western blotting results (**C**) and the summary of changes normalized with Gapdh (**D**). Open and black bars represent the control and VCR groups, respectively [N = 3 per group per sex] Mean ± SE, fold difference (the control group taken as 1), *p < 0.05, **p < 0.005 vs. the control group. Figures were prepared using Adobe Photoshop CS6 and GraphPad Prism 8.4.3 (https://www.graphpad.com/scientific-software/prism/).

**Figure 6:**
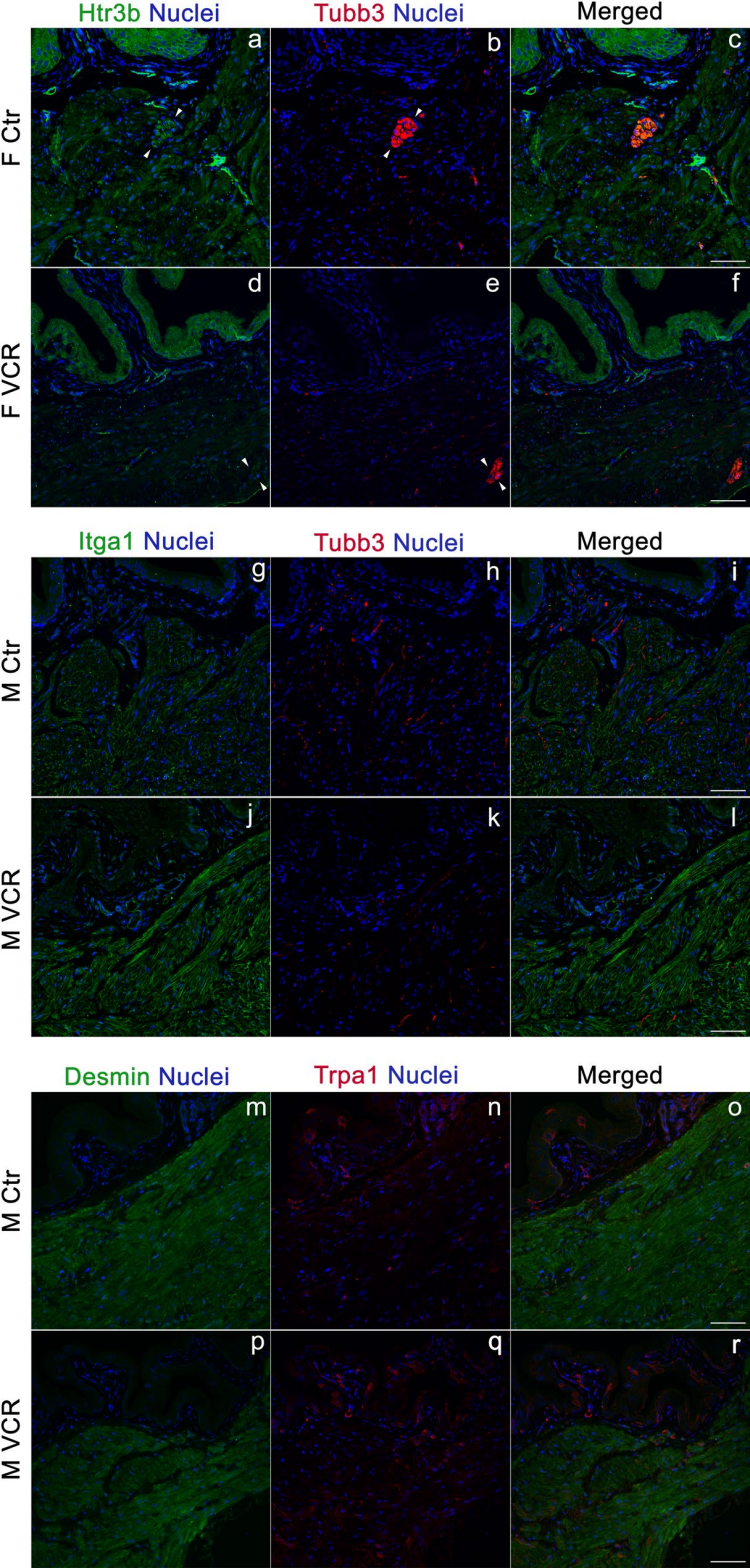
Immunofluorescence labeling of the bladders. Representative immunohistochemical images with antibodies against Htr3b (green, **a** and **d**), Tubb3 (red, **b** and **e**), and merged image (**c** and **f**) along nuclei staining using DAPI (blue) on the bladder sections from female mice in the control (**a**–**c**) and VCR (**d**–**f**) groups. Arrowheads indicate the cluster-forming Tubb3 IR. Representative immunohistochemical images on the bladder sections from male mice in the control (**g**–**i** and **m**–**o**) and VCR (**j**–**l** and **p**–**r**) groups with antibodies against Itga1 (green, **g** and **j**), Tubb3 (red, **h** and **k**), Desmin (green, **m** and **p**), Trpa1 (red, **n** and **q**) and merged image (**i**, **l**, **o**, and **r**) along nuclei staining using DAPI (blue). Scale bars = 250 µm. Figures were prepared using Adobe Photoshop CS6.

**Figure 7:**
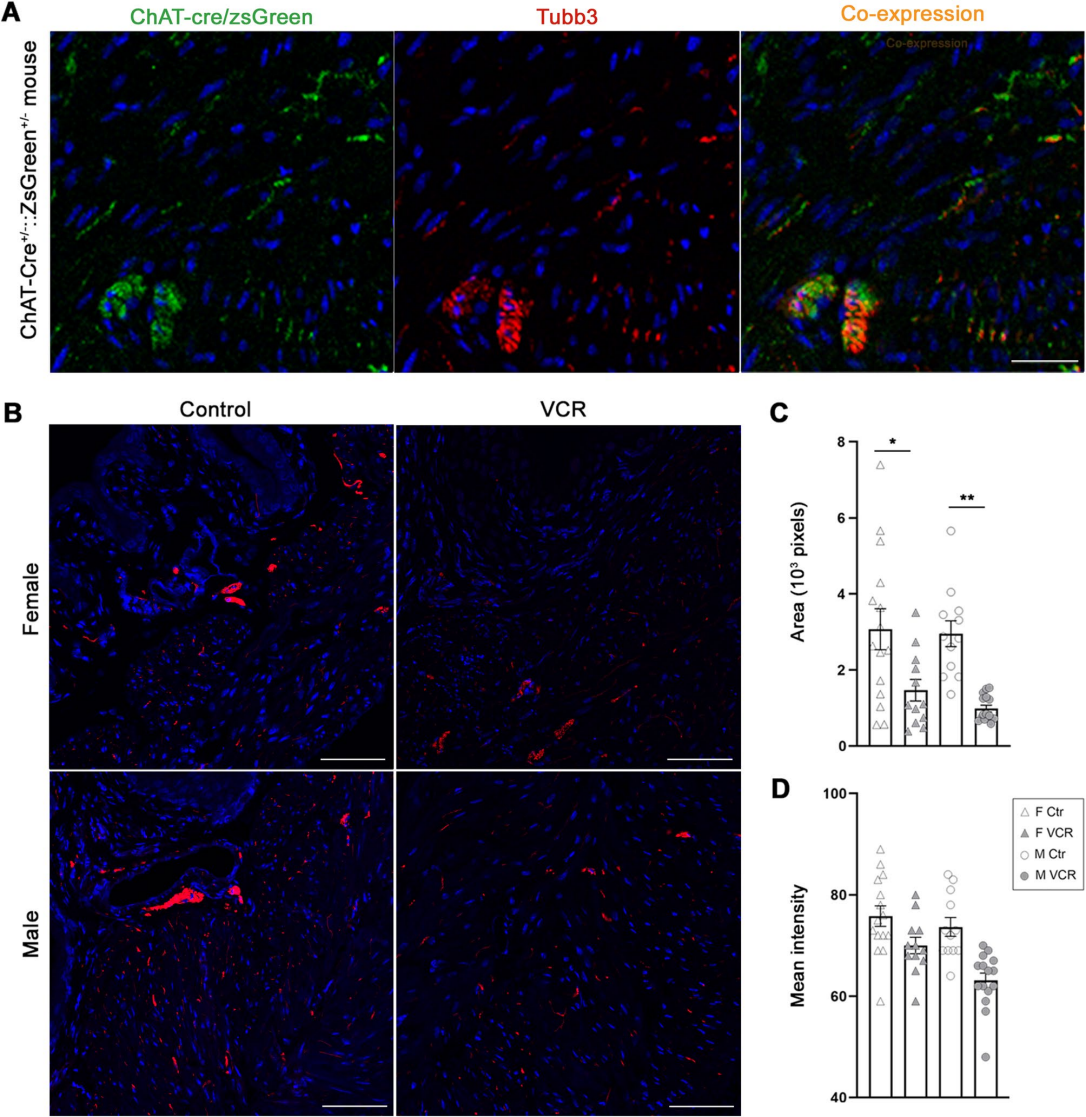
Tubb3 immunofluorescence labeling. (**A**) representative immunofluorescent labeling images with antibody against Tubb3 (red) of the bladder sections from ChAT-Cre^+/−^:: ZsGreen^+/−^ mice, in which the expression of ZsGreen protein (green) were restricted in ChAT-expression cells. The merged images of ZsGreen and Tubb3 IR (right), shows Tubb3 IR in the Zs-Green positive cells in cluster forms [N = 3] (**B**), representative images of Tubb3 IR (red) in the bladders from female (upper) and male (lower) from the control (left) and VCR (right) groups. Cell nuclei were stained with DAPI (blue) [N = 4–5 per group per sex]. Scale bars = 20 µm (**A**) and 250 µm (**B**). The area (**C**) and mean intensity (**D**) measurements for Tubb3-IR in the bladder sections. *p < 0.05, **p < 0.005. Figures were prepared using Adobe Photoshop CS6 and GraphPad Prism 8.4.3 (https://www.graphpad.com/scientific-software/prism/).

To explore the molecular mechanisms contributing to VCR-induced changes in LUT function, qPCR analyses were performed for the genes associated with neuromuscular control of the LUT function, bladder overactivity as well as inflammatory signaling using samples from the urinary bladders and Ls-DRG. In addition, previously reported CIPN-associated genes (*Cep72*, *Foxc1*, *Itga1*, *Nmnat2*, *Sarm1* and *Vac14*) were examined^10,11^. The bladder qPCR results showed that VCR induced a significant downregulation of serotonin receptor 3β (*Htr3b*) in females, and significant elevations of 3 genes: *Trpa1*, *IL-2* and *Itga1* in males (Fig. 5A). Western blotting revealed a decreased level of Htr3b in female and elevated expression of IL-2 and Itga1 in male mice exposed to VCR at the protein level (Fig. 5C,D). The Ls-DRG qPCR demonstrated that VCR induced a female-specific downregulation of *Cav1.2* and male-specific upregulations of four genes, *P2*×*4* and *P2*×*7*, *IL-2* and *CD68* (Fig. 5B). There was a male-specific tendency towards increased expression of cytokines associated with neuroinflammation IL-1β, IL-6, and Tnfα in the Ls-DRG (1.5–1.9-fold, p = 0.06–0.12). Pkc_Ɛ_ has been suggested to play a role in VIPN specifically in males^12^; however, no differences in the expression level of Pkc_Ɛ_ were detected in the bladder and Ls-DRG between the groups. Htr3b immunoreactivity (IR) was detected in the cytoplasm of the urothelial cells, fibroblasts, and the DSM cells in the bladders from female mice in the control group (Fig. 6a–c). In the lamina propria and DSM layers, the Htr3b IR were also seen in cluster-forming structures (arrowheads in Fig. 6a). In comparison, Htr3b IR in female mice in VCR group were more subtle, especially in the DSM cells and cluster-forming structures (Fig. 6d–f). A neuronal-specific tubulin β Class III (Tubb3) IR was detected in either fiber-like or punctate patterns in the lamina propria and DSM layers alongside the cells (Fig. 6b,e,h,k). Intense Tubb3 IR were observed in cluster-like structures within the lamina propria and DSM layers which appear to be mainly consist of ChAT-expressing neurons (Fig. 7A). Htr3b IR was colocalized with cluster forming Tubb3 IR in the control group, while it was faint in the bladders from female mice exposed to VCR (Fig. 6e). Itga1 IR were detected primarily on the cell surface of DSM in male bladders, and the signals were more intense in mice received VCR than the control group (Fig. 6g and i vs. j and l). The Tubb3 IR in cluster forms were observed more often in the control group than in VCR group in the bladder sections which manifested as a significant decrease in the area of Tubb3 IR in both female and male mice, suggesting that VCR induced a decreased incorporation of Tubb3 protein in nerve fibers in the bladder (Fig. 7B–D). Western blotting showed the comparable level of total β-tubulin protein in the bladder, suggesting a compensatory incorporation of non-Tubb3 β-tubulin isotypes in bladder innervation following VCR exposure. The Trpa1 IR were observed in a subset of the urothelial cells and blood vessels as well as in small punctate or fiber-like patterns in the lamina propria and DSM layers in the bladder from males from each group (Fig. 6n,q). The image analyses revealed an increased Trpa1 IR in the bladders from VCR group compared to the controls (the area, 1.7-fold, p = 0.001 and mean intensity, 1.1-fold, p = 0.021), indicating an elevated level of Trpa1 protein similar to the qPCR result. Desmin IR was detected in the detrusor myocytes at comparable level in both groups of male mice (Fig. 6m,o,p,r).

## Discussion

The findings of this study indicate that systemic VCR exposure during the juvenile period induced: (1) decreased urinary frequency accompanied with increases in functional bladder capacity, (2) female-specific increase in intravesical pressure at micturition, (3) male-specific detrusor overactivity (4) mechanical hyposensitivity, and (5) sex dimorphic changes in gene expression patterns in the bladder and Ls-DRG in mice.

Normal LUT function is designed to store and expel urine in a coordinated, controlled manner. This relies on the tissue components’ (the muscles and mucosa) function and regulation by the central and peripheral nervous systems. Hence, any chemotherapy effects on the neurons could have adverse effects on LUT function. VCR is an important part of many pediatric oncology treatment regimens; however, it is known to cause impairment of axonal transport, distal axonopathy and neuroinflammation, leading to VIPN^1^. Previously two clinical cases of the neurotoxic effects of VCR on LUT function; urinary bladder paralysis and detrusor areflexia, were reported^13,14^. In this study, the mice who received VCR demonstrated a decrease in micturition frequency and increased NVCs. Such findings are similar to a clinical trend we have observed in a recent prospective study of decreased micturition frequency and significantly higher rate of overactive bladder symptoms in pediatric cancer survivors who received VCR and/or doxorubicin (DOX) therapy when compared to healthy control subjects^3^. In the current study of our murine model, VCR exposure also induced an increase in functional bladder capacity without affecting the voiding efficiency, indicating that VCR had no major impacts on the elastic and contractile properties of the DSM and sphincters, and the synergistic coordination between them. Our in vitro physiology study provided evidence that VCR exposure did not change the DSM contractile responses evoked by facilitating the neurotransmitter release (EFS) and their signaling pathways (CCh and αβMeATP) or depolarization of DSM cells (KCl). These results suggest that the DSM and efferent nerves in the bladder retained their function to contract and empty the bladder. The mice in VCR group exhibited a reduced susceptibility to mechanical stimuli, one of the VIPN symptoms reported in children^15^, suggesting an impairment of the afferent pathway in these animals. We therefore speculate that the VCR-induced sensory dysfunction caused a reduction of bladder filling sensation, leading to delays in recognizing bladder fullness and the urge to urinate, which manifested as a decreased frequency of voiding and increased functional bladder capacity.

We identified female-specific downregulation of Htr3b following VCR exposure in the bladder. Htr3 receptors are assembled in homomeric α subunits (Htr3a) or heteromeric complex formed by α and β subunits (Htr3a/b). It has been shown that Htr3a/b receptor displays a larger single channel conductance and response amplitude than Htr3a homomer receptor, and Htr3b subunit facilitates the stable expression of the Htr3a/b receptors on the cell membrane^16,17^. Lines of evidence suggest that Htr3 plays a pivotal role in bladder afferent excitability, and inhibition of Htr3 increased bladder capacity under both normal and pathological conditions^18–20^. In the Ls-DRG, we observed a female-specific downregulation of *Cav1.2* gene which has been shown to play an important role in pain processing, neuropathy-associated mechanical hypersensitivity^21^. Altogether, we consider that the VCR-induced downregulations of Htr3b compromises Htr3a/b channel activity in the bladder coinciding with a decrease in *Cav1.2* in the Ls-DRG, thus impairing the bladder afferent firing and causing decreased bladder sensation in female mice. As such, the animals did not experience urges to urinate until the bladder was filled to the maximum capacity, which resulted in the increases in intravesical pressure and functional bladder capacity following VCR exposure. The increased NVCs without detrusor overactivity in vitro observed in VCR-treated female mice appears to be another sign that the bladder was reaching maximum capacity. Meanwhile, VCR exposure induced an elevation of IL-2, Itga1 and Trpa1 in the bladder and *P2*×*4*, *P2*×*7*, *IL-2 and CD68* in the Ls-DRG in male mice. IL-2 has pleiotropic immune functions; acts as an immunostimulatory and immunoregulatory cytokine. Studies demonstrated an elevated IL-2 expression in neuron and glia in patients with degenerative neurological disorders, suggesting that IL-2 plays a role in neuroinflammatory pathways and the disease process^22,23^. CD68 is a common marker for activated macrophage lineage cells including microglia in central nervous system and associated with neurodegenerative disorders^24^. The robust nerve injury-induced macrophage expansion in the lumbar DRG and its contribution to mechanical allodynia has been reported in males but to a much less extent in female mice^25^. Accumulating evidence implicates that P2x4 potentiates P2x7-dependent release of proinflammatory cytokines including IL-1β, IL-6 and Tnfα in both central and peripheral nervous systems, leading to neuroinflammation, which contributes to induce neuropathy and abnormal nociception^26^. Studies demonstrated that CD68, P2x4 and P2x7 are expressed in the satellite glial cells (SGC) and macrophages in sensory ganglia, and that the DRG macrophages and SGCs contribute to nerve fiber damage, nerve injury induced neuropathic pain and CIPN^27–30^. We observed a male-specific trend of upregulation of IL-1β, IL-6 and Tnfα in the Ls-DRG following VCR exposure. Therefore, we consider that VCR induced inflammatory responses at least partly through the P2x4-P2x7 pathway in the Ls-DRG in male mice, which disturbed the bladder sensation similar to the mechanical hyposensitivity observed in the von Frey tests. Such hyposensitivity led to increases in the functional bladder capacity and bladder compliance in male mice. Itga1 is expressed in a wide variety of cells including immune cells, fibroblasts, and smooth muscle cells. Studies suggested that Itga1-expressing tissue resident immune cells produce high levels of proinflammatory cytokines and IL-2 and IL-15 stimulate their cytotoxic potential^31,32^. As such, Itga1 may be involved in VCR-induced neuroinflammation in the bladder in male mice. Future experiments should be conducted to test this hypothesis. Integrin α1β1 receptors (heterodimers of Itga1 and β1 subunit) serve as cell-surface receptor for collagen IV and laminin, involved in cell–cell adhesion, inflammation, and fibrosis. Studies showed that Itga1 was induced in smooth muscle cells upon differentiation from myogenic progenitor cells^33^, and Itga1 ligands promoted survival and a contractile phenotype of smooth muscles^34,35^. Based on the data that male mice who received VCR tended to have increased bladder size despite significantly reduced growth compared to the control group and increased expression on DSM cells, we speculate that Itga1 upregulation promotes survival and maintaining contractile function of DSM cells in the male bladder. Trpa1 channels expression was reported in the urothelial and DSM cells and bladder afferent nerves. Urothelial Trpa1 channels facilitate release of neurotransmitters including ATP and acetylcholine which activate receptors on afferent nerve terminals, interstitial cells, and DSM cells^36^. Studies demonstrated that Trpa1 plays a major role in detrusor overactivity as well as mechanosensory and nociceptive dysfunction in pathological states, particularly, with inflammation^37,38^. We surmise that the VCR-induced inflammation responses enhanced Trpa1 expression and signaling, resulting in detrusor overactivity in male mice.

The cumulative dose of 4 mg/kg in mice is calculated as an equivalent dose of 12 mg/m^2^ in children^39^, which is a relatively low dosage for pediatric cancer therapy^40^. Yet, even at this dose, the mice showed signs of LUTD. The clinical use of higher dose of VCR, as well as its use with other chemotherapy agents such as DOX, can cause more severe VIPN and other side effects including constipation and a syndrome of inappropriate secretion of antidiuretic hormone (SIADH)^41,42^. Our previous studies demonstrated that both systemic DOX exposure and functional constipation induce LUTD in juvenile mice^8,43^, suggesting an increased risk of LUTD in children treated with VCR and DOX treatment.

**Figure 8:**
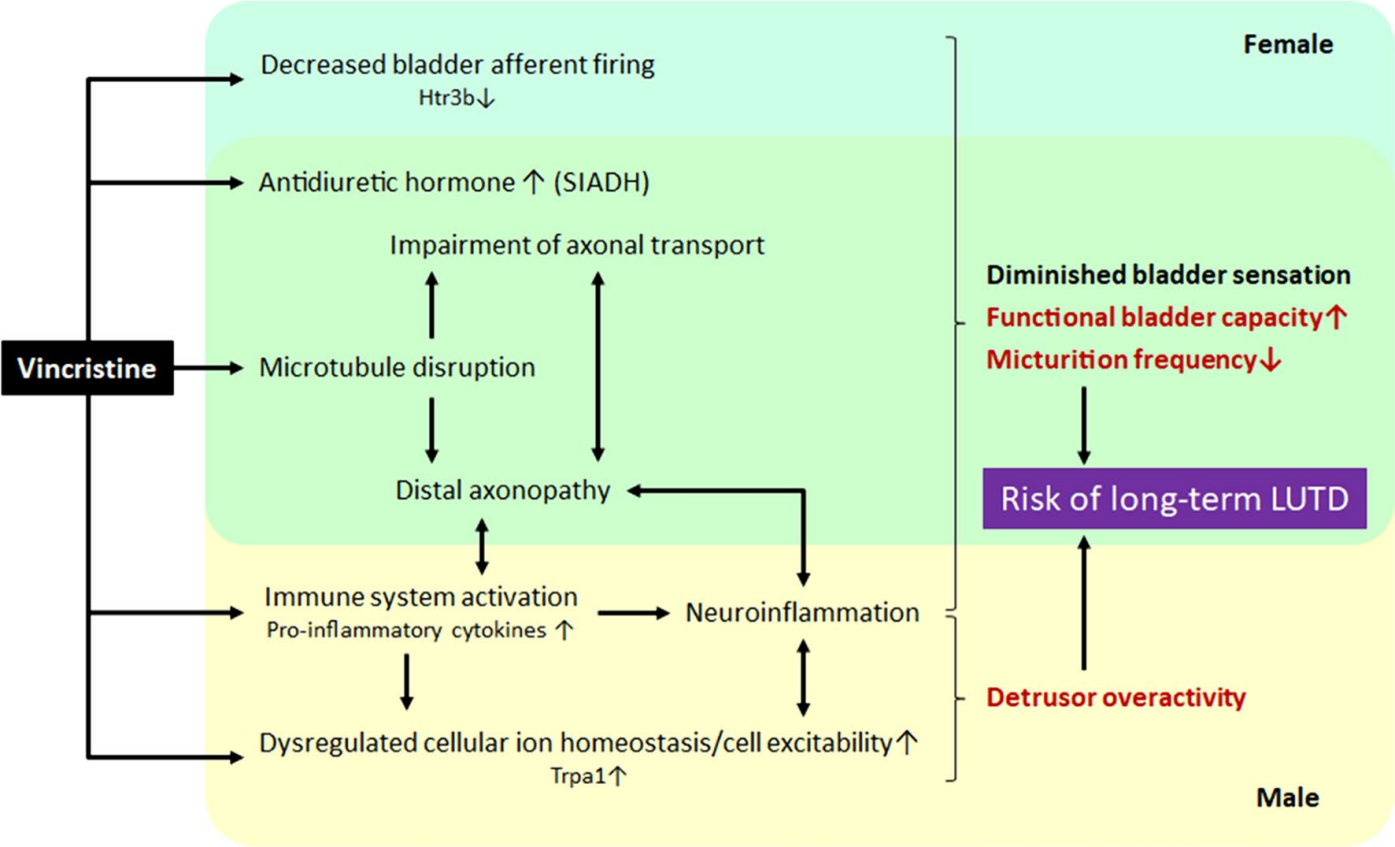
Schematic diagram of the proposed mechanisms underlying vincristine induced alterations in lower urinary tract function. Systemic VCR exposure induces deficiency in bladder sensation, leading to a decrease in micturition frequency accompanied with an increased bladder capacity. In addition to the VCR-induced impairments of axonal transport and distal axonopathy, sexual dimorphic mechanisms contribute to the phenotype; in females, a decrease in bladder afferent firing through downregulation of Htr3b signaling while in males, neuroinflammation. Syndrome of inappropriate secretion of antidiuretic hormone (SIADH) can exacerbate the symptoms. In males, the dysregulations of cellular ion homeostasis in the bladder through enhanced Trpa1 signaling induce increases in cell excitability, leading to detrusor overactivity. The VCR-induced neuroinflammation also can play a role in detrusor overactivity in male. Systemic VCR exposure can increase risk of developing long-term sex specific LUTD via multiple mechanisms.

Collectively, our data revealed that systemic VCR exposure during the juvenile period affects genes and molecular pathways in the bladder and Ls-DRG in a sexual dimorphic pattern, which lead to sex-specific side effects. A schematic presentation of our proposed mechanisms underlying VCR-induced alterations in LUT function is shown in Fig. 8. VCR-induced changes might clinically present as gender-specific signs of LUTD, and follow-up urological assessment may be warranted in pediatric cancer patients treated with VCR.

## Supplementary Information

Supplementary Figures.

Supplementary Tables.
