# Supplementary material for: Sexual dimorphic impacts of systemic vincristine on lower urinary tract function
Source: Sci Rep. 2022 Mar 24;12:5113. doi: 10.1038/s41598-022-08585-3 (PMC8948262; doi:10.1038/s41598-022-08585-3)
Supplement: Supplementary file 1 — Supplementary Figures. [file 41598_2022_8585_MOESM1_ESM.pdf]

Supplementary Figure S1. Full size Western Blot images using the bladder samples from each group. The bladders from female mice with anti-Htr3b antibody (A); with anti-β-tubulin and anti-Gapdh (B, upper). The bright field image of the blot (B, lower). The bladders from male mice with anti-Sma and anti-IL-2 (C); with anti-Itga1 (D); with anti-Pkcε (E); and with anti-β-tubulin and anti-Gapdh (F).

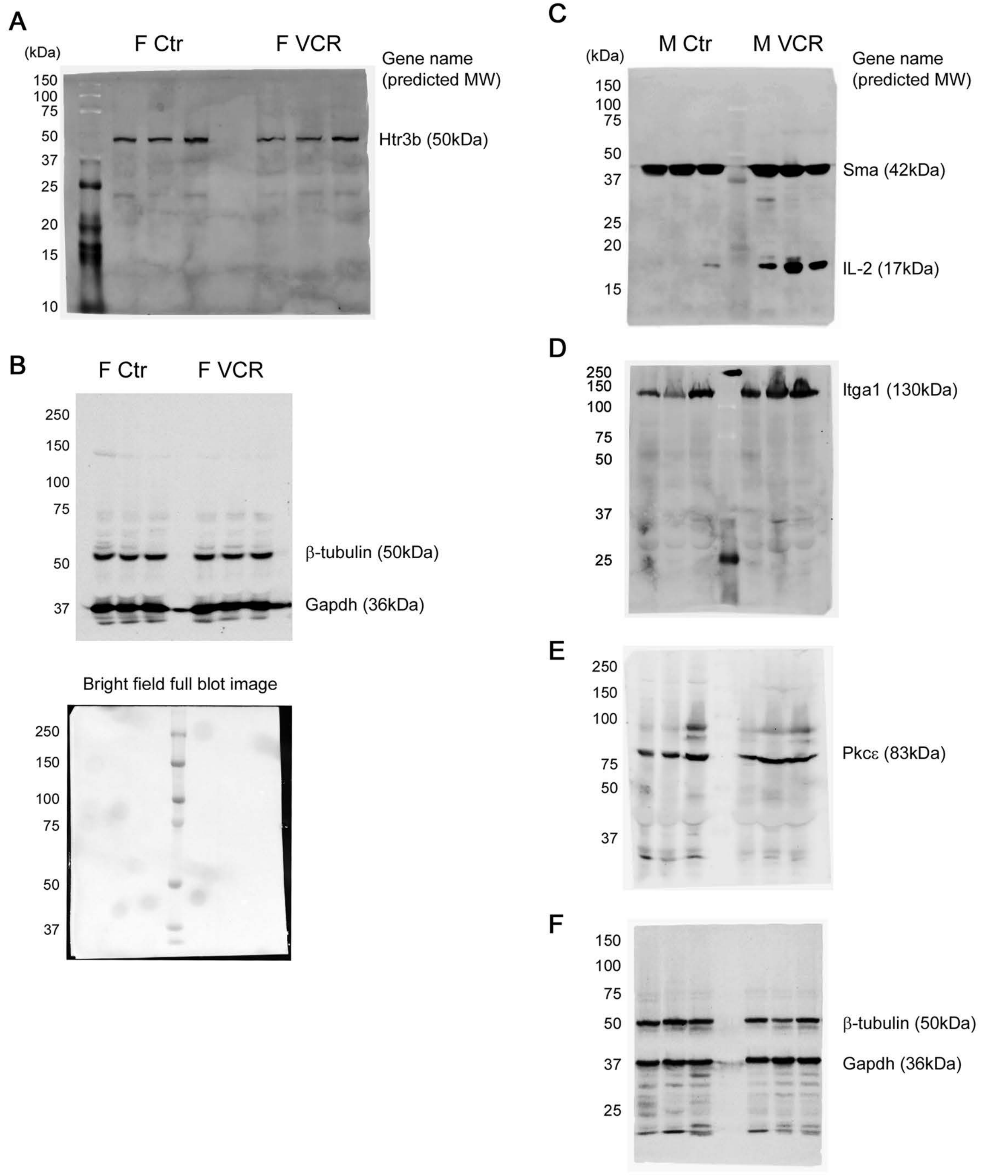

Supplementary Figure S2. Validation of specificity of antibodies. A. Western blotting with antibodies against IL-2 (left) or Htr3b (center) using protein lysates from cell lines, or Itga1 using protein samples from mouse tissues (right). B, immunofluorescence images with antibodies against Tubb3 (red) and Trpa1 (green) on the lumbar DRG (top) and kidney (bottom)

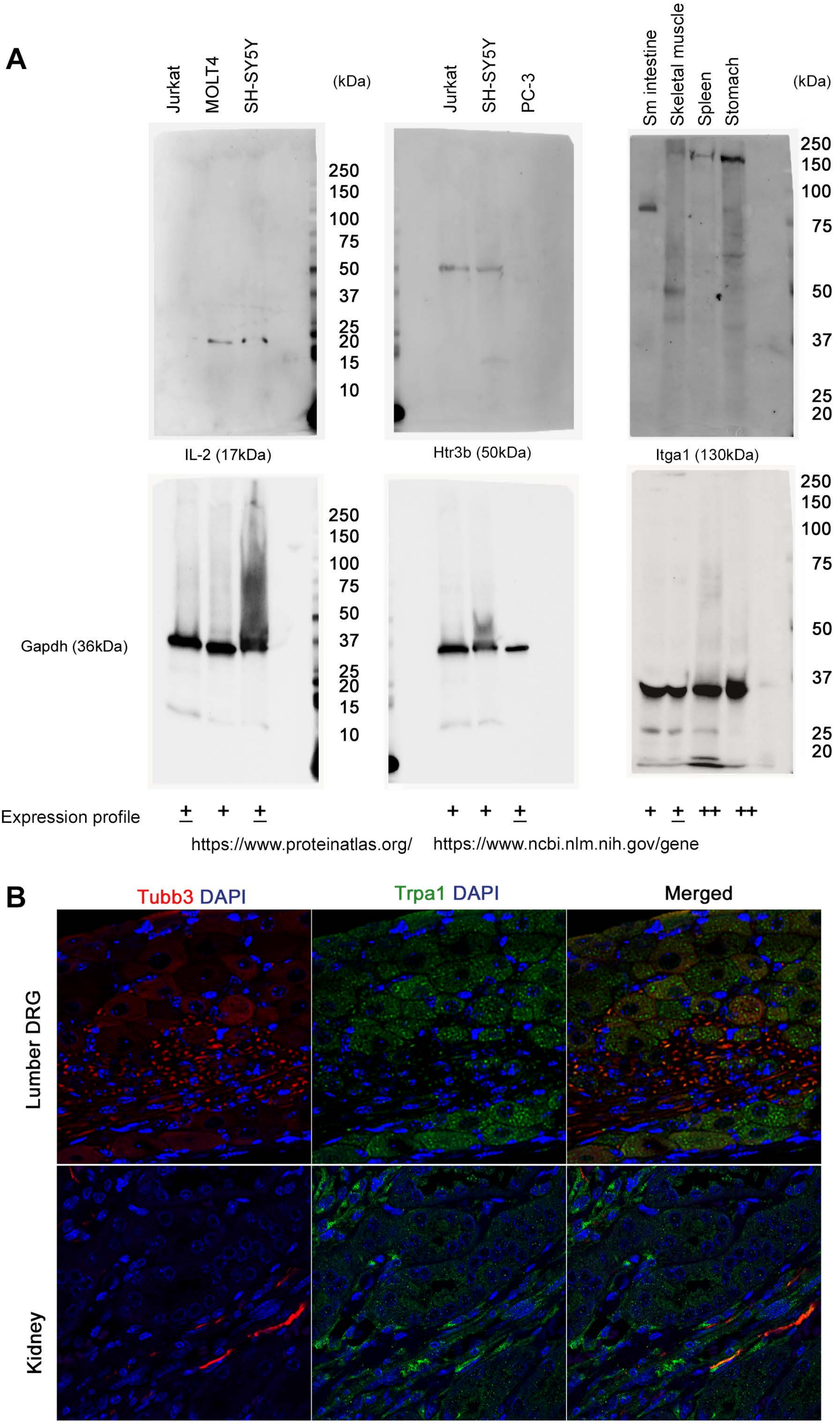

Comparable IR in doi.org/10.1136/gut.2008.175901 (DRG), doi.org/10.3390/ijms22052309 (kidney)
