# Supplementary material for: Sexual dimorphic impacts of systemic vincristine on lower urinary tract function
Source: Sci Rep. 2022 Mar 24;12:5113. doi: 10.1038/s41598-022-08585-3 (PMC8948262; doi:10.1038/s41598-022-08585-3)
Supplement: Supplementary file 2 — Supplementary Tables. [file 41598_2022_8585_MOESM2_ESM.docx]

**Supplementary Table S1. Primer sequences**

| Gene ID |  | 5'-3' sequence | Accession # |
| --- | --- | --- | --- |
| Adra2 | F | CTGGCTGAGATCATGTGACTAC | NM_007417.4 |
|  | R | CCTTCCACAGTCTGCCTAAA |  |
| Adrb2 | F | AATAGCAACGGCAGAACGGA | NM_007420 |
|  | R | CTTCCTTGGGAGTCAACGCT |  |
| Adrb3 | F | GTTGTCCTGGTGTGGATCGT | NM_013462 |
|  | R | CATAGGGCATGTTGGAGGCA |  |
| Bdnf | F | CTGAGCGTGTGTGACAGTATTA | NM_001048139.1 |
|  | R | CTTTGGATACCGGGACTTTCTC |  |
| Cav1.2 | F | CTACCTGCTCATCCCTTTCTTC | NM_009781.4 |
|  | R | TTTCACCTCTGTTCCCTTCAC |  |
| Cav1.3 | F | CGTTGGTCCTGTCTACAACTAC | NM_001302637.1 |
|  | R | GATGACGAAGCCCACAAAGA |  |
| Cep72 | F | CAGAGTGCTGTCTCCTAAACTC | NM_028959.3 |
|  | R | CCACTTGAGCACCTGTCTATC |  |
| Chat | F | GAGACCTCATCTGTGGAGTTTG | NM_009891.2 |
|  | R | GGCCTCTAGCTCTTTCCTTTG |  |
| Cd68 | F | CCCACCTGTCTCTCTCATTTC | NM_001291058.1 |
|  | R | GTATTCCACCGCCATGTAGT |  |
| Chrm2 | F | TGGTTTGGCTATTACCAGTCCT | NM_203491.3 |
|  | R | CTGAAGGTGGCGGTTGACTT |  |
| Chrm3 | F | CCTCGCCTTTGTTTCCCAAC | NM_033269.4 |
|  | R | TTGAGGAGAAATTCCCAGAGGT |  |
| Foxc1 | F | GGGAGATGGCGGTTTGATTA | NM_008592.2 |
|  | R | TCTAGATAGGAGCGGCAGATAG |  |
| Gapdh | F | AACAGCAACTCCCACTCTTC | NM_001289726.1 |
|  | R | CCTGTTGCTGTAGCCGTATT |  |
| Htr3a | F | TCTGTCTCTCTGACTTCCCTATC | NM_001099644 |
|  | R | GGTTAGAGGGTACTTGGTTTCTC |  |
| Htr3b | F | AATGTGCTGGTGGGCTATAC | NM_020274.4 |
|  | R | CCATGCAGACGGTAAAGAAGA |  |
| Htr4 | F | TCGGCATAGTTGATGTGATAGAG | NM_008313.4 |
|  | R | CACAGAGCAGGTGATAGCATAG |  |
| Htr7 | F | GGAACAGAAAGCAGCCACTA | NM_008315.3 |
|  | R | CAGGAGGTGCCACAGATAAA |  |
| Itga1 | F | GGAACTCAGGAAAGGAGGATTC | NM_001033228.3 |
|  | R | GAGTCCTGAAAGTCGTGCTTAT |  |
| IL-2 | F | GCGGCATGTTCTGGATTTG | NM_008366.3 |
|  | R | TGTGTTGTCAGAGCCCTTTAG |  |
| IL-6 | F | GCCTTCTTGGGACTGATGCT | NM_001314054.1 |
|  | R | GACAGGTCTGTTGGGAGTGG |  |
| IL-10 | F | CCAAGACCAAGGTGTCTACAA | NM_010548.2 |
|  | R | GGAGTCCAGCAGACTCAATAC |  |
| Kcnma1 | F | GCATTGGTGCCCTCGTAATA | NM_001253358.1 |
|  | R | GCCAAAGTAGAGGAGGAAGAAC |  |
| Kcnmb4 | F | GTGAACAACTCCGAGTCCAA | NM_021452.1 |
|  | R | TCTCTTACAGGGCGGGATATAG |  |
| Ngf | F | ACAGCCACAGACATCAAGGG | NM_001112698 |
|  | R | GTGTGAGTCGTGGTGCAGTA |  |
| Nmnat2 | F | GTAGTCTTGATGCTCTCCTGTG | NM_175460.3 |
|  | R | CTGCCTTCTTTCCTGGATGT |  |
| nNos | F | ACCAGCACCTTTGGCAATGGAG | NM_008712.3 |
|  | R | GAGACGCTGTTGAATCGGACCT |  |
| Pgk1 | F | CACAGAAGGCTGGTGGATTT | NM_008828.3 |
|  | R | CTTTAGCGCCTCCCAAGATAG |  |
| Pkcε | F | GCTCGGAAACACCCTTATCT | NM_011104.3 |
|  | R | ACATGAGGTCTCCACCATTTAC |  |
| P2x2 | F | CAAGTATGACCCTGCCTCTTC | NM_001164833.1 |
|  | R | CCCATAGGCTTTGATGAGAGTT |  |
| P2x3 | F | CCAGCTTCAGCAGGAAATAGA | NM_145526.2 |
|  | R | GAGGACCTCATGGGAGAATAAAG |  |
| P2x4 | F | TGGGTGTTCGTGTGGGAAAA | NM_011026 |
|  | R | TTGGTCACAGCCACACCTTT |  |
| P2x7 | F | GGATGGACCCACAGAGCAAA | NM_001038845 |
|  | R | CCAGGCAGAGACTTCACAGG |  |
| Sarm1 | F | TTCCTCCTACCGTCCATCTT | NM_001168521.1 |
|  | R | CATCTGCCTCACCTCAGAATAG |  |
| TBP | F | GGGATTCAGGAAGACCACATAG | NM_013684.3 |
|  | R | CCTCACCAACTGTACCATCAG |  |
| Tac1 | F | AAGCGGGATGCTGATTCCTC | NM_009311 |
|  | R | TCTTTCGTAGTTCTGCAT |  |
| Tac2 | F | TCCCTGCTTCGGAGACTCTAC | NM_001199971 |
|  | R | TGTCACGTTTCTGTGGAAGTG |  |
| Trpa1 | F | CACAGACCG ACTAGATGAAGAAG | NM_001348288.1 |
|  | R | CAGGAGGATGTCAGCATTGT |  |
| Trpm8 | F | CTCCTGCTGTTTGCCTATGT | NM_134252 |
|  | R | CATCACAGAAGAGGACGAAGAC |  |
| Trpv1 | F | CCGGCTTTTTGGGAAGGGT | NM_001001445.2 |
|  | R | GAGACAGGTAGGTCCATCCAC |  |
| Trpv2 | F | TTCAGGATGGGGTCAATGCC | NM_011706 |
|  | R | GCCTCGGTAGAACTCATCGG |  |
| Trpv4 | F | CCTATGGGCCTGTGTATTCTTC | NM_022017.3 |
|  | R | CGGTTCTCGATCTTGCTGTT |  |
| Tubb3 | F | TAGACCCCAGCGGCAACTAT | NM_023279.3 |
|  | R | GTTCCAGGTTCCAAGTCCACC |  |
| Tnfα | F | CTGAGTTCTGCAAAGGGAGAG | NM_013693.3 |
|  | R | CCTCAGGGAAGAATCTGGAAAG |  |
| Vac14 | F | CGCAGAGTTATCCTCTGATGTG | NM_146216.3 |
|  | R | CAGGTTCTTAGGCTCCTTGTG |  |
| Vegfa | F | GCACATAGAGAGAATGAGCTTCC | M95200.1 |
|  | R | CTCCGCTCTGAACAAGGCT |  |
| Vegfr1 | F | TAAGCCTGGGGAACTCATTCT | AK005502.1 |
|  | R | CCAAAGATGCGACTGTAATGCTG |  |

**Supplementary Table S2. Number of animals in each assay.**

| Sex | Treatment | Von Frey | Cystometry | Bladder | | | | Ls-DRG  qPCR |
| --- | --- | --- | --- | --- | --- | --- | --- | --- |
|  |  |  |  | **Histology** | **Contractility** | **qPCR** | **WB** |  |
| F | **Saline** | 4 | 4 | 4 | 6 | 5 | 3 | 4 |
|  | **VCR** | 3 | 5 | 4 | 6 | 4 | 3 | 4 |
| M | **Saline** | 6 | 6 | 5 | 6 | 5 | 3 | 5 |
|  | **VCR** | 7 | 6 | 5 | 6 | 4 | 3 | 4 |

qPCR, Quantitative Real-time polymerase chain reaction; WB, Western blotting
